# Rising gasoline prices increase new motorcycle sales and fatalities

**DOI:** 10.1186/s40621-015-0054-3

**Published:** 2015-09-17

**Authors:** He Zhu, Fernando A. Wilson, Jim P. Stimpson, Peter E. Hilsenrath

**Affiliations:** 1 College of Public Health, University of Nebraska Medical Center, 984350 Nebraska Medical Center, Omaha, NE 68198-4350 USA; 2 School of Public Health, City University of New York, 2180 Third Avenue, New York, NY 10035 USA; 3 Joseph M. Long Chair in Healthcare Management and Professor of Economics, Eberhardt School of Business, Thomas J. Long School of Pharmacy and Health Sciences, University of the Pacific, 3601 Pacific Avenue, Stockton, CA 95211 USA; 4 School of Medicine, Duke University, 201 Trent Dr, Durham, NC 27710 USA

**Keywords:** Motorcycles, Mortality, Traffic accidents

## Abstract

**Background:**

We examined whether sales of new motorcycles was a mechanism to explain the relationship between motorcycle fatalities and gasoline prices.

**Methods:**

The data came from the Motorcycle Industry Council, Energy Information Administration and Fatality Analysis Reporting System for 1984–2009. Autoregressive integrated moving average (ARIMA) regressions estimated the effect of inflation-adjusted gasoline price on motorcycle sales and logistic regressions estimated odds ratios (ORs) between new and old motorcycle fatalities when gasoline prices increase.

**Results:**

New motorcycle sales were positively correlated with gasoline prices (*r* = 0.78) and new motorcycle fatalities (*r* = 0.92). ARIMA analysis estimated that a US$1 increase in gasoline prices would result in 295,000 new motorcycle sales and, consequently, 233 new motorcycle fatalities. Compared to crashes on older motorcycle models, those on new motorcycles were more likely to be young riders, occur in the afternoon, in clear weather, with a large engine displacement, and without alcohol involvement. Riders on new motorcycles were more likely to be in fatal crashes relative to older motorcycles (OR 1.14, 95 % confidence interval (CI) 1.02–1.28) when gasoline prices increase.

**Conclusions:**

Our findings suggest that, in response to increasing gasoline prices, people tend to purchase new motorcycles, and this is accompanied with significantly increased crash risk. There are several policy mechanisms that can be used to lower the risk of motorcycle crash injuries through the mechanism of gas prices and motorcycle sales such as raising awareness of motorcycling risks, enhancing licensing and testing requirements, limiting motorcycle power-to-weight ratios for inexperienced riders, and developing mandatory training programs for new riders.

**Electronic supplementary material:**

The online version of this article (doi:10.1186/s40621-015-0054-3) contains supplementary material, which is available to authorized users.

## Background

Motorcycle safety has received much attention from both researchers and policymakers, especially because motorcycle-related fatalities and injuries are increasing in the United States at the same time that overall motor vehicle fatalities have been decreasing (National Center for Injury Prevention and Control 2012; Oster and Strong 2013). According to the National Highway Traffic Safety Administration (NHTSA), motorcyclist fatalities increased every year from 1994 to 2008; in 2008, 5312 motorcyclist fatalities accounted for 14 % of total motor vehicle deaths (National Highway Traffic Safety Administration 2014a). Additionally, the total cost of motorcycle-related fatalities and injuries has reached US$12 billion per year (Naumann et al. 2010).

There are several reasons that may contribute to this increasing trend of motorcycle-related fatalities and injuries. First, motorcycles have higher crash risk than passenger cars. Motorcyclists are about 30 times more likely to die than passenger car occupants for every mile travelled (National Highway Traffic Safety Administration 2012). Furthermore, an increasing number of motorcycle registrations and motorcycle miles traveled have increased risk exposure for the riders. In prior studies, increasing gasoline prices over time were associated with higher numbers of motorcyclist fatalities and injuries, possibly resulting from people substituting toward motorcycling from other motor vehicles in order to lower their fuel costs (Hedlund 2013; Morris 2009; Wilson et al. 2009). It was estimated that motorcycle fatalities would have been over 50 % lower if gasoline prices had not increased from 1998 to 2007 (Wilson et al. 2009). Hyatt et al. (2009) suggested that gasoline prices were positively associated with population-adjusted rates of motorcycle injuries and fatalities, although there was no significant association after adjusting for the number of registered motorcycles on the road. Zhu et al. (2015) found that motorcycle fatal and non-fatal injuries were highly correlated with increasing gasoline prices for the state of California.

Although prior studies have implied that increasing gasoline prices may encourage people to substitute toward new motorcycles, leading to more motorcycle-related crashes, the relationship between trends in gasoline prices and sales of new motorcycles and consequent fatalities of new motorcycles has not been examined to our knowledge. New motorcycle sales are more likely to be associated with less experienced riders who may be entering motorcycling in response to escalating gasoline prices. In fact, one study found newer motorcycles to have a higher number of fatalities per 10,000 motorcycles sold compared to older motorcycles (Paulozzi 2005). Our study aims to explore the association of gasoline prices with industry-provided data on new motorcycle sales, and consequently motorcycle fatalities. In addition, we used a census database of all fatal motorcycle crashes on US public roads in order to characterize the relationship of gasoline prices with fatalities on new versus older motorcycle models.

## Methods

Annual motorcycle sales were defined as new on-highway model units sold in the United States. These data were compiled in the Motorcycle Industry Council’s (MIC) Statistical Annual report based on annual sales data provided by motorcycle manufacturers (MIC 2010). The MIC is the national trade association of motorcycle manufacturers in the United States. A total of 11,736,000 new on-highway motorcycles were sold from 1984 to 2009. Off-road motorcycles (e.g., dirt bikes) were excluded from the analysis because they are largely used for recreational purposes and, thus, are unlikely to be viable alternatives for persons considering the purchase of motorcycles as an alternative to a car or truck.

Retail gasoline prices were provided by the United States Energy Information Administration (EIA) (Energy Information Administration 2013), which includes the annual and monthly average gasoline price per gallon for all grades in the United States. EIA collects gasoline pump prices for all grades (regular, medium, and premium) by telephone from approximately 800 retail gasoline outlets every Monday, and this sample was drawn from about 115,000 self-serve retail gasoline outlets. Price data were compiled using weighted average prices at city, state, regional, and national levels based on the number of pumps, sales volume, grades, and geographic areas. All prices were adjusted to 2009 dollars using the Consumer Price Index (CPI) (Bureau of Labor Statistics 2013). Use of the CPI to adjust gasoline prices for inflation is consistent with prior studies (Hyatt et al. 2009; Wilson et al. 2009; Grabowski and Morrisey 2004).

Data on motorcycle fatalities were retrieved from the National Highway Traffic Safety Administration (NHTSA)’s Fatality Analysis Reporting System (FARS) (National Highway Traffic Safety Administration 2014a). The FARS database is a nationwide census recording every fatal motor vehicle crash on public roadways that resulted in at least one fatally injured occupant within 30 days (National Highway Traffic Safety Administration 2013). The FARS provides detailed characteristics of the occupants, motorcycle model and year, and crash factors. We examined all motorcycle fatalities except those associated with three-wheel motorcycles, off-road motorcycles, and unknown motorcycle types (National Highway Traffic Safety Administration 2013). Total motorcycle fatalities include all motorcycle-related occupant (riders and passengers) fatalities in crashes involving at least one motorcycle. New motorcycle fatalities were defined as the number of motorcyclist fatalities with motorcycles having a model year in the same or prior year as the crash (e.g., a 2007 or 2008 model would be defined as new if the crash occurred in 2007). Similarly, older motorcycle fatalities were categorized as fatalities occurring on 1–3-year-old motorcycle models and 4 or more year-old models. There were 86,706 motorcyclist fatalities in the 26-year period from 1984 to 2009, and crashes on new motorcycles accounted for 10.5 % (9,105) of these, 1–3-year-old motorcycles for 29.8 % (25,841), 4 or more year-old motorcycles for 58.6 % (50,808). Motorcycle models in which the year was unknown were dropped from the analysis (1.1 % (952)).

Data on the characteristics of fatal motorcycle crashes were also obtained from the FARS (NHTSA). Demographic characteristics of fatal motorcyclists included occupant type (motorcycle rider and passenger), gender (male and female), and age (<16, 16–20, 21–29, 30–44, 45–64, and 65 and more years old). At the crash level, day of week was categorized into weekday (Monday to Friday) and weekend (Saturday and Sunday); hour of day included 00:00–5:59, 6:00–11:59, 12:00–17:59, and 18:00–23:59; weather, which may affect visibility and road condition, was depicted as clear weather (no adverse atmospheric conditions) and not clear weather (including rain, sleet, snow, fog, etc., and their combinations). At the motorcycle level, factors included: (1) whether the motorcycle rider was reported by law enforcement as having alcohol involvement (drinking vs no drinking); (2) whether the motorcycle rider has a valid license (valid vs. not valid); (3) motorcycle engine displacement size (<125, 125–449, 450–749 cc, and 750 cc+); and (4) use of a helmet or not. We also include inflation-adjusted average annual disposable personal income, annual average precipitation (inches), and temperature (degrees in Fahrenheit) as control variables (U.S. Bureau of Economic Analysis 2015; National Climatic Data Center Climate Data Online 2014), because these may significantly affect the decision to purchase and ride a motorcycle (Hedlund 2013; Wilson et al. 2009).

We examined the period 1984 to 2009, corresponding to the availability of new motorcycle sales data from the MIC at the time of this study. Sales data were only available at an annual level, thus, we analyzed annual data for the United States. Autoregressive Integrated Moving Average (ARIMA) time-series models were used to estimate the effect of inflation-adjusted gasoline prices on annual number new motorcycle sales and number of sales per 100 million population for 1984–2009. ARIMA models have been widely developed to analyze time-series data in previous traffic safety studies (Friedman et al. 2007; Martinez-Schnell and Zaidi 1989; Quddus 2008; Ramstedt 2008). Prior studies of gasoline prices and motorcycle safety have used ARIMA models to estimate the association of gasoline prices on motorcycle outcomes (Hyatt et al. 2009; Wilson et al. 2009; Zhu et al. 2015). ARIMA models are also appropriate to address serial correlation in the data (Becketti 2013; Box et al*.*^2011^; Box-Steffensmeier et al*.*^2014^).

The format of model is ARIMA(*p*,*d*,*q*): *p* is the number of autoregressive (AR) terms; *q* is the number of moving-averaging (MA) terms; and *d* is the number of non-seasonal differences. We first checked the stationarity of the data by Dickey-Fuller tests to determine that the difference should be 1 (*d* = 1). Secondly, we examined correlograms to identify AR (*p* = 1) and MA (*q* = 2) terms. Third, the parameters were estimated by using the specified model ARIMA(1,1,2) with the above control variables. Fourth, the white noise *Q* test of the residual was applied to diagnose the adequacy of the model. Additionally, the number of deaths associated with new motorcycles was divided by the total annual new motorcycle sales to calculate new motorcycle model fatalities per 100,000 new motorcycle sales for each year, and ARIMA results were combined with new motorcycle fatality rates to estimate the impact of the new sales resulting from changing gasoline prices on motorcycle fatalities.

Finally, referring to new motorcycle and associated deaths, we described the characteristics of fatally injured motorcycle occupants and crashes at the individual level, and statistical significance was examined by using a chi-squared test. Monthly gasoline price data were merged into FARS data by linking with time of crash, and we conducted logistic regressions to ascertain the likelihood that new-versus-older model motorcyclists would be fatally injured in a crash when inflation-adjusted gasoline prices increased. All statistical analyses were performed by Stata 13.0 (Stata Corp, College Station, TX, USA 2013).

## Results

**Fig. 1:**
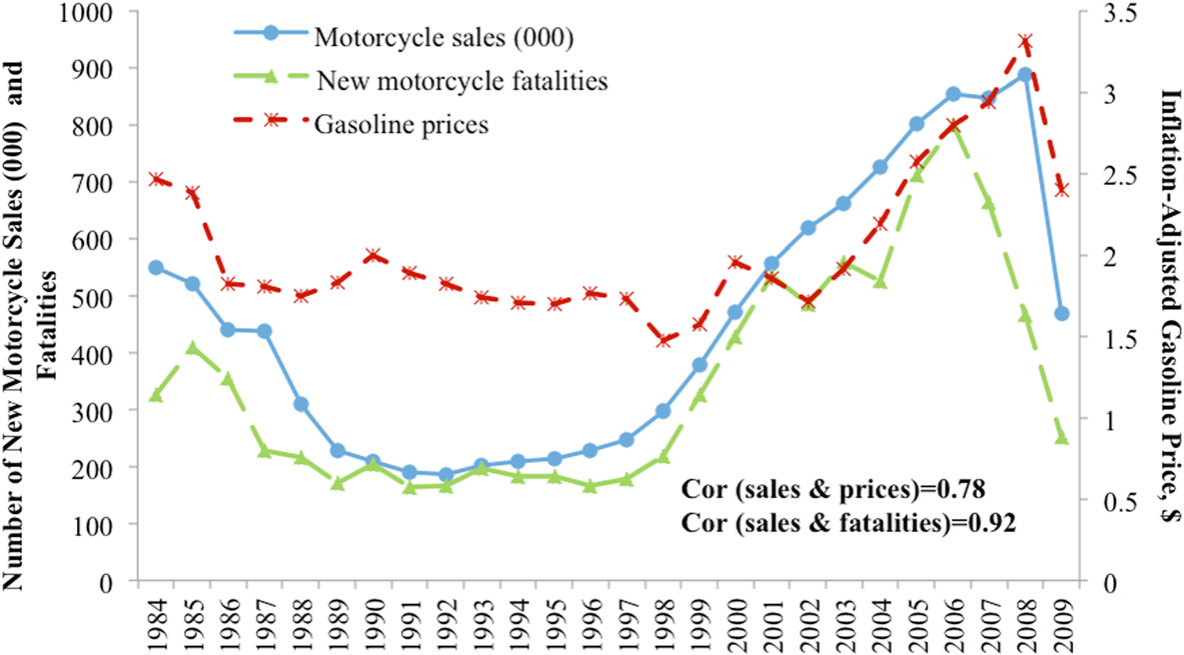
Inflation-adjusted gasoline prices, annual number of new motorcycle sales and new motorcycle fatalities: US, 1984–2009. *Cor* correlation

Figure 1 describes the annual new motorcycle sales and inflation-adjusted gasoline prices for the period 1984–2009. New motorcycles had their lowest sales with 186,000 units in 1992 and, afterward, grew every year until 2008, reaching 888,000 units—a 377 % increase. Gasoline prices decreased to less than $2 at the end of the 1990s but began to rise after 2002, reaching $3.32 in 2008. Both sales and gasoline prices reversed their gains in 2009. Trends in new motorcycle sales and gasoline prices are highly correlated (*r* = 0.78), although sales lagged the decrease in gasoline prices in 2006–2007. For example, the price elasticity of gasoline prices on motorcycle sales was 1.84, which means a 10 % increase in gasoline prices is associated with a 18.4 % increase in new motorcycle sales. Figure 1 also shows that the number of new motorcycle fatalities tracked closely with the fluctuation in number of motorcycle sales. The annual correlation between new motorcycle fatalities and sales was 0.92, although the trends in fatalities and sales temporarily diverged in 1988 and 2002. From 1984 to 2009, annual new motorcycle fatalities per 100,000 motorcycle sales on average is 78.9, which is higher than the rate of all motorcycle fatalities per 100,000 motorcycle registrations (65.5) (see Additional file 1: Table S1).

We used ARIMA regression modeling to predict the impact of changing gasoline prices on sales and, consequently, deaths from new motorcycle purchases (Table 1). The descriptive statistics of the variables can be found in Additional file 2: Table S2. ARIMA regression modeling predicts that for every US$1 increase in gasoline prices, there are an additional 295,000 new motorcycle sales in the US. (*p* < 0.001)—or an additional 103 new motorcycle sales per 100,000 population (*p* < 0.001) (see Additional file 3: Table S3). Meanwhile, inflation-adjusted disposable personal income and precipitation also have a positive association with new motorcycle sales, while temperature has a negative association; however, these coefficients were not statistically significant. Correspondingly, this increase of motorcycle sales was estimated to be associated with 233 additional fatalities associated with new motorcycle models annually, which was computed by multiplying 295,000 (estimated additional sales) and 78.9 (annual new motorcycle fatalities per 100,000 sales reported above). For example, from 1998 to 2008, inflation-adjusted gasoline prices increased by US$1.85, and our ARIMA regression analysis suggests that this increase in gasoline prices—and the consequent increase in new motorcycle sales—were associated with 431 additional deaths on new motorcycle models. Other model specifications such as log transformation of the measures did not substantively change our findings.

In Table 2, the characteristics of motorcycle occupants and crashes were compared among three motorcycle model age groups. There were 85,754 fatal motorcycle occupants with non-missing motorcycle model year from 1984 to 2009. Generally, among new motorcycle models, occupants tended to be riders instead of passengers (92.0 %), younger (49.9 % for 16–29 years old), male (91.2 %) and riding on weekdays (62.9 %) and afternoons (36.4 % between 12:00 and 17:59), and in clear weather (97.5 %). Interestingly, compared to riders of motorcycle models more than 3 years old, riders of new models were less likely to have alcohol involvement (29.7 vs. 42.7 %), wear a motorcycle helmet (59.3 vs. 49.4 %), have a valid license (85.1 vs. 78.2 %), and be riding motorcycles with larger engine displacements (60 vs. 56.5 % with 750 cc+ displacement).

**Table 1:** ARIMA regression-predicated impact of inflation-adjusted gasoline price on motorcycle sales and related new motorcycle fatalities

Gasoline prices (US$)	Mean annual motorcycle sales	Mean annual new motorcycle fatalities
Actual numbers		
2.04^a^	451,000	350
Predicted impact		
1.50	291,000	229
2.00	439,000	345
2.50	587,000	462
3.00	734,000	578
3.50	882,000	695

^a^Average annual inflation-adjusted gasoline price for the study period, 1984–2009. ARIMA also adjusted for average disposable income per capita, precipitation in inches, and temperature in degrees

**Table 2:** Characteristics of fatal occupants and their motorcycles and crashes among three groups of motorcycle-model ages

	Motorcycle model ages^a^				
	Total	Less than 1 year	1–3 years	4 years and more	*P* value
Number of motorcyclists	85,754	9,105	25,841	50,808	
Occupant type					
Riders	91.1	92.0	91.5	90.7	<0.001
Passengers	8.9	8.0	8.5	9.4	
Gender					
Male	91.3	91.2	91.3	91.3	0.907
Female	8.7	8.8	8.7	8.7	
Age					
<16	1.3	1.0	1.3	1.3	<0.001
16–20	12.4	15.2	14.0	11.1	
21–29	32.3	34.7	34.7	30.6	
30–44	32.0	27.5	28.4	34.7	
45–64	19.9	19.9	19.6	20.0	
65 and more	2.1	1.7	2.0	2.3	
Day of week					
Weekday	62.7	62.9	62.6	62.8	0.794
Weekend	37.3	37.1	37.4	37.2	
Hour of day					
00:00–5:59	16.8	15.3	16.3	17.4	<0.001
6:00–11:59	11.5	12.2	12.5	10.9	
12:00–17:59	33.5	36.4	34.7	32.4	
18:00–23:59	38.2	36.1	36.5	39.4	
Weather					
Clear	96.9	97.5	97.1	96.7	<0.001
Rain, snow, or fog	3.1	2.5	2.9	3.3	
Helmet use					
Used	52.5	59.3	56.1	49.4	<0.001
Not used	47.5	40.7	43.9	50.6	
Rider alcohol involvement					
Drinking	38.8	29.7	34.5	42.7	<0.001
No drinking	61.2	70.3	65.6	57.3	
Rider license status					
Valid	80.4	85.1	83.2	78.2	<0.001
Not valid	19.6	14.9	16.8	21.8	
Motorcycle engine displacement					
<125 cc	1.2	0.9	1.2	1.2	<0.001
125–449 cc	7.2	4.1	5.0	9.0	
450–749 cc	33.9	35.0	34.6	33.4	
750 cc+	57.7	60.0	59.2	56.5	

^a^Excludes 952 motorcycle occupants who have missing values on model age for their motorcycles from all fatally injured motorcyclists

We also examined the associations between gasoline prices and motorcycle model age among fatal motorcycle crashes using logistic regressions. The odds ratio (OR) indicated that when the gasoline prices increased US$1, a fatally injured motorcyclist had higher odds of riding a new rather than an older motorcycle model, adjusting for state and year (OR 1.14 (95 % confidence interval (CI) 1.02–1.28) (see Additional file 4: Table S4). This suggests that changing gasoline prices tend to impact the distribution of fatal motorcycle injuries toward new motorcycle models compared to older models.

## Discussion

Results from our study show a strong relationship between new motorcycle sales and gasoline prices since the mid-1980s. This expands upon prior research showing a positive relationship between gasoline prices and motorcycle fatalities (Hyatt et al. 2009; Wilson et al. 2009; Zhu et al. 2015). Our study suggests that people may respond to rising gasoline prices by purchasing motorcycles due to their fuel economy instead of traveling by automobile. According to the NHTSA, motorcycle miles per gallon is two or three times higher than for passenger cars on average (Federal Highway Administration 2014). Moreover, compared to most automobiles, the pricing of motorcycles may be more attractive. For instance, the average price of a new on-highway motorcycle was less than US$12,000 in 2009 using MIC statistics (MIC 2010). This compares to nearly $30,000 for a new automobile in the same year (USA TODAY 2013). Furthermore, our study findings show that increased sales resulting from rising gasoline prices was associated with fatal crashes involving new model motorcycles. Compared to crashes involving older motorcycles, riders of new motorcycles tended to be younger with crashes occurring in the afternoon. Alcohol involvement was also less prevalent among riders of new motorcycles relative to older motorcycles.

Our results showing the importance of gasoline prices on new motorcycle sales are a conservative measure of the overall impact of prices on ridership. Wilson and colleagues estimated that each dollar increase in gasoline prices resulted in about 1500 additional motorcycle fatalities, which includes crashes involving both old and new motorcycles (Wilson et al. 2009). We estimated that a dollar increase in gasoline prices would result in 295,000 new motorcycle sales and, consequently, 233 fatalities on new motorcycles. This suggests that the predominant impact of gasoline prices may be to increase motorcycling among existing riders instead of increasing the number of new riders. This is not surprising because there are approximately eight million registered motorcycles in the United States. However, the fatality rate is likely to be higher for new riders.

Several studies report that individuals tend to purchase newer or more fuel-efficient cars in response to high gasoline prices (Austin 2008; Gillingham 2011; Jeihani and Sibdari 2010; McCarthy 1996; Feng et al*.*^2005^; Klier and Linn 2010; Kahn 1986). High gasoline prices also influence people to use other alternative transportation, such as walking, bicycles, carpools, buses and subways (American Public Transportation Association 2011). Other literature demonstrates a negative association between driving and gasoline prices (Grabowski and Morrisey 2004; Sen et al. 2014). The overall net impact of gasoline prices on fatalities and injuries associated with fuel-efficient but smaller vehicles and use of alternative transportation modalities is uncertain. However, substitution toward motorcycling in response to rising gasoline prices would be expected to increase crashes due to the substantially higher risk of injury from riding (Lin and Kraus 2009). For example, NHTSA reports that motorcyclists are about 30 times more likely to die than passenger car occupants for every mile travelled (National Highway Traffic Safety Administration 2012). About two in every five fatally injured motorcyclists were involved in single vehicle collisions (National Highway Traffic Safety Administration 2007). This suggests that a large number of riders may not be properly trained or experienced in riding motorcycles. The pioneering study on motorcycle safety by Hurt and colleagues based on 900 in-depth crash investigations of motorcycle riders found that 92 % of riders involved in crashes were self-taught or had learned to ride from friends and family (Hurt et al. 1981). Furthermore, one-third of riders in crashes had taken no evasive actions to avoid the collision, and a large proportion of the evasive actions that were used were determined to have been inappropriate (e.g., not using both front and rear brakes) (Hurt et al. 1981).

There are no restrictions on the type of motorcycle that may be purchased by adult riders in the United States. In addition, few states have mandatory training requirements for all new motorcycle riders (American Motorcyclist Association AMA 2015). About 60 % of states will waive one or more testing requirements if a rider completes a rider education course. This situation contrasts with licensing in the European Union, for example, where motorcycle licenses are graduated based on motorcycle power, age, and years of experience (European Commission 2013). Therefore, there are few legal barriers limiting motorcycle power-to-weight ratios or mandating basic skills training for inexperienced riders in the United States. Our results imply that new motorcycles involved in fatal crashes tended to have larger engine displacements compared to older motorcycles. One previous study also reported that over 50 % of motorcycle fatalities in 2003 were accounted by new motorcycle sales between 2000 and 2003 (Paulozzi 2005).

The high fatality rate of motorcycles compared to passenger cars found by NHTSA may also result from lack of safety equipment (National Highway Traffic Safety Administration 2012). Data from the National Occupant Protection Use Survey suggest that only 60 % of motorcyclists use a DOT-compliant helmet (National Highway Traffic Safety Administration 2014b). Motorcycle helmet use laws have been shown to be effective in decreasing risk of injury and death (Grabowski and Morrisey 2004; Bavon and Standerfer 2010; Mertz and Weiss 2008; Houston and Richardson 2007; Muller 1980, 2004; Auman et al*.*^2002^). However, only 19 states have universal helmet laws, and no state requires the use of any other protective gear (Governors Highway Safety Association 2015). Motorcycle safety gear can be costly, both in an absolute sense and as a percentage of the price of a scooter or motorcycle. Thus, it is uncertain whether individuals motivated to purchase motorcycles for their fuel economy place sufficient importance on the purchase of appropriate motorcycle safety gear. In fact, our data show that only 59 % of fatally injured riders on new motorcycle models wore a helmet. Interestingly, this percentage is higher than that for riders of older motorcycles (49 % for riders of motorcycles 4 years or older). This suggests that the already low rate of helmet use among purchasers of new motorcycles continues to decrease over time.

A possible policy-based solution to increasing the safety of motorcycling for new riders may be incentivizing them to take formal training on riding motorcycles (French et al. 2009). Six states (Florida, Oregon, Rhode Island, Connecticut, Texas and Maine) currently require motorcycle training for new adult licensees (American Motorcyclist Association AMA 2015). Two recent articles reviewed existing studies assessing effectiveness of motorcycle training programs, and they reported mixed findings on their effectiveness ((Daniello et al*.*^2009^; Kardamanidis et al. 2010). For example, protective equipment use was found to increase after training. However, high attrition rates in training classes were also a limitation in their effectiveness. Although most states waive some testing requirements if riders have taken a training course, there is substantial variation in the administration, content, and length of training programs across states (Daniello et al*.*^2009^; Kardamanidis et al. 2010). Thus, effective motorcycle training interventions should be developed and implemented to improve the riders’ skills and reduce crashes. There have been substantial efforts by organizations such as the Motorcycle Safety Foundation to provide standardized training programs across the country (Buche 2004).

This study has limitations. First, the FARS database does not provide data on non-fatal injuries. However, we expect injuries to be positively correlated with fatalities in motorcycle crashes. Although we have sales data for new motorcycles, there are no comparable data for used motorcycles. Thus, the impact of gasoline prices on the demand for motorcycles will be conservative. A small sample size (*n* = 26) was used in this study, and monthly or state-level data were not available on motorcycle sales. In the literature, there is no consistent standard for the minimum sample size of ARIMA models. It is typically recommended to have a minimum sample size of 50 observations (Box and Pierce 1970; McCleary et al. 1980). However, a minimum sample size of 36 was suggested by Makridakis et al. (1998). Linden (2015) argued that long time-series data were required for forecasting, and the ARIMA model can be applied for smaller sample sizes. The fit of the model may also be a more important consideration than the sample size. Gasoline prices may differentially impact miles traveled on new versus old motorcycle models; however, data on motorcycle miles traveled stratified by new versus old motorcycle models are not available. FARS does not provide information on how experienced riders were at the time of the crash. We believe purchasers of new motorcycles may consist largely of inexperienced riders but will also include a significant share of experienced riders who wish to either return to motorcycling or upgrade their existing motorcycle. Finally, there are other variables that impact motorcycle fatality rates that cannot be analyzed using FARS. One that has received recent attention is lane-splitting where motorcycles advance between lanes, often in heavy traffic. It is possible that this would differentially impact crash risk for newer versus older riders.

## Conclusions

To our knowledge, this study is the first to examine the association of trends in sales of new motorcycles with changing gasoline prices. Although prior research shows a negative relationship between gasoline prices and driving, we present evidence that gasoline prices may increase incentives to purchase motorcycles, leading to an increase in fatalities from motorcycle crashes. Raising awareness of motorcycling risks, enhancing licensing and testing requirements, limiting motorcycle power-to-weight ratios for inexperienced riders, and mandatory training programs for new riders may help to reduce crashes from the increased popularity of motorcycling.

## Additional files

Additional file 1: Table S1.
**Trends of gasoline prices, motorcycle sales, and fatalities from 1984 to 2009.**
Additional file 2: Table S2.
**Descriptive statistics of the measures.**
Additional file 3: Table S3.
**ARIMA(1,1,2) regression estimated impact of inflation-adjusted gasoline prices on new motorcycle sales.**
Additional file 4: Table S4.
**Odds ratios of a fatality associated with a new versus older motorcycle model from logistic regression.**
