# Supplementary material for: Rising gasoline prices increase new motorcycle sales and fatalities
Source: Inj Epidemiol. 2015 Sep 17;2(1):23. doi: 10.1186/s40621-015-0054-3 (PMC5005806; doi:10.1186/s40621-015-0054-3)
Supplement: Additional file 1: Table S1. — Trends of gasoline prices, motorcycle sales, and fatalities from 1984 to 2009. [file 40621_2015_54_MOESM1_ESM.doc]

Table S1: Trends of gasoline prices, motorcycle sales and fatalities from 1984 to 2009

| **Year** | **Price of Gasoline ($)** | **Motorcycle sales (000s)** | **Total motorcycle fatalities** | **Total Motorcycle fatalities per 100,000 motorcycle registration** | **Fatalities from new motorcycles** | **New motorcycle fatality rate per 100,000 motorcycle sales** |
| --- | --- | --- | --- | --- | --- | --- |
| 1984 | 2.47 | 550 | 4,431 | 80.9 | 326 | 59.3 |
| 1985 | 2.38 | 520 | 4,417 | 81.1 | 409 | 78.7 |
| 1986 | 1.82 | 440 | 4,309 | 81.9 | 355 | 80.7 |
| 1987 | 1.81 | 438 | 3,834 | 78.0 | 227 | 51.8 |
| 1988 | 1.74 | 310 | 3,492 | 76.2 | 215 | 69.4 |
| 1989 | 1.83 | 227 | 3,036 | 68.5 | 170 | 74.9 |
| 1990 | 2.00 | 208 | 3,129 | 73.5 | 203 | 97.6 |
| 1991 | 1.89 | 190 | 2,703 | 64.7 | 163 | 85.8 |
| 1992 | 1.82 | 186 | 2,291 | 56.4 | 167 | 89.8 |
| 1993 | 1.74 | 201 | 2,336 | 58.7 | 197 | 98.0 |
| 1994 | 1.70 | 210 | 2,190 | 58.3 | 183 | 87.1 |
| 1995 | 1.70 | 214 | 2,114 | 54.2 | 183 | 85.5 |
| 1996 | 1.76 | 228 | 2,046 | 52.8 | 166 | 72.8 |
| 1997 | 1.73 | 247 | 2,028 | 53.0 | 177 | 71.7 |
| 1998 | 1.47 | 298 | 2,186 | 56.3 | 219 | 73.5 |
| 1999 | 1.58 | 379 | 2,374 | 57.2 | 325 | 85.8 |
| 2000 | 1.95 | 471 | 2,783 | 64.0 | 427 | 90.7 |
| 2001 | 1.85 | 556 | 3,077 | 62.8 | 536 | 96.4 |
| 2002 | 1.71 | 618 | 3,150 | 62.9 | 484 | 78.3 |
| 2003 | 1.92 | 662 | 3,583 | 66.7 | 558 | 84.3 |
| 2004 | 2.19 | 725 | 3,827 | 66.3 | 526 | 72.6 |
| 2005 | 2.57 | 801 | 4,418 | 70.9 | 710 | 88.6 |
| 2006 | 2.79 | 855 | 4,679 | 70.1 | 799 | 93.5 |
| 2007 | 2.93 | 846 | 4,986 | 69.8 | 663 | 78.4 |
| 2008 | 3.32 | 888 | 5,060 | 65.3 | 465 | 52.4 |
| 2009 | 2.40 | 468 | 4,227 | 53.3 | 252 | 53.8 |
