# Supplementary material for: Rising gasoline prices increase new motorcycle sales and fatalities
Source: Inj Epidemiol. 2015 Sep 17;2(1):23. doi: 10.1186/s40621-015-0054-3 (PMC5005806; doi:10.1186/s40621-015-0054-3)
Supplement: Additional file 2: Table S2. — Descriptive statistics of the measures. [file 40621_2015_54_MOESM2_ESM.doc]

Table S2: Descriptive statistics of the measures

| Measures | Obs. | Mean | Std. Dev. | Min | Max |
| --- | --- | --- | --- | --- | --- |
| Number of new motorcycle sales per 100 million population | 26 | 451 | 234 | 186 | 888 |
| Inflation-adjusted gasoline prices | 26 | 2.04 | 0.46 | 1.47 | 3.32 |
| Inflation-adjusted disposable personal income (000s) | 26 | 29.15 | 4.50 | 22.48 | 36.08 |
| Precipitation (inches) | 26 | 2.56 | 0.16 | 2.16 | 2.82 |
| Temperature (degrees in Fahrenheit) | 26 | 52.90 | 0.83 | 51.26 | 54.25 |
