# Supplementary material for: Rising gasoline prices increase new motorcycle sales and fatalities
Source: Inj Epidemiol. 2015 Sep 17;2(1):23. doi: 10.1186/s40621-015-0054-3 (PMC5005806; doi:10.1186/s40621-015-0054-3)
Supplement: Additional file 3: Table S3. — ARIMA(1,1,2) regression estimated impact of inflation-adjusted gasoline prices on new motorcycle sales. [file 40621_2015_54_MOESM3_ESM.doc]

**Table S3**: ARIMA(1,1,2) regression estimated impact of inflation-adjusted gasoline prices on new motorcycle sales

| **Outcomes** | **The number of new motorcycle sales (000s)** | **P-values** | **New motorcycle sales per 100,000 population** | **P-values** |
| --- | --- | --- | --- | --- |
| Coefficient of inflation-adjusted gasoline prices | 295.44 | <0.001 | 102.57 | <0.001 |
| Coefficient of inflation-adjusted disposable personal income (000s) | 7.46 | 0.633 | 0.30 | 0.486 |
| Coefficient of precipitation | 40.80 | 0.301 | 18.51 | 0.113 |
| Coefficient of temperature | -7.29 | 0.306 | -1.87 | 0.367 |
| N | 25 | | 25 | |
