# Supplementary material for: Rising gasoline prices increase new motorcycle sales and fatalities
Source: Inj Epidemiol. 2015 Sep 17;2(1):23. doi: 10.1186/s40621-015-0054-3 (PMC5005806; doi:10.1186/s40621-015-0054-3)
Supplement: Additional file 4: Table S4. — Odds ratios of a fatality associated with a new versus older motorcycle model from logistic regression. [file 40621_2015_54_MOESM4_ESM.doc]

**Table S4**: Odds ratios of a fatality associated with a new versus older motorcycle model from logistic regression

|  | **Variable** | **OR** | **95% CI** | **P-value** | |
| --- | --- | --- | --- | --- | --- |
| **Outcome: New motorcycle-related fatality (1) versus Older motorcycle-related fatality(0)** | | | | | |
| Model I | Inflation-adjusted gasoline price | 1.17 | [1.13,1.22] | | <0.001 |
| Model II* | Inflation-adjusted gasoline price | 1.14 | [1.02,1.28] | | 0.019 |

*Notes:* *State- and year-effects were controlled.
